# Prevalence of herpes simplex virus 2 among MSM in Mainland China: a systematic review and meta-synthesis

**DOI:** 10.1186/s12981-022-00469-w

**Published:** 2022-10-01

**Authors:** Mingming Shi, Xiao Zhang, Mengqing Chen

**Affiliations:** 1 Center for Disease Control and Prevention of Shangcheng District, Hangzhou, Zhejiang China; 2 Department of Health Development, Affiliated Hangzhou First People’s Hospital, Zhejiang University School of Medicine, Hangzhou, Zhejiang China

## Abstract

**Background:**

As one of the most prolific sexually transmitted infections (STIs) in the world, *Herpes Simplex Virus Type 2* (HSV-2) is one of the primary causes of genital ulcers. In addition, HSV-2 infection multiplies the risk of acquiring HIV. Men who have sex with men (MSM) are at particularly high risk of contracting both diseases. Unfortunately, little information is available with regarding to the comprehensive prevalence of HSV-2 among MSM in mainland China. The objective of this manuscript was to determine the composite prevalence of HSV-2 among MSM in mainland China via systematic review and meta-synthesis.

**Methods:**

We systematically searched PubMed, Embase, Chinese National Knowledge Infrastructure, WanFang Database for Chinese Periodicals, and the VIP Database for Chinese Technical Periodicals for relevant articles published from the database’s inception to 28 April 2022 that reported data on the prevalence of HSV-2 within the MSM population in mainland China. We considered publications to be eligible for inclusion if they satisfied these conditions: (1) publication participants were MSM in China mainland. Studies were excluded if participants were exclusively all HIV-positive MSM, all HIV-negative MSM, injection-drug users, or MSM sex workers. These studies would have introduced selection bias and skewed pooled prevalence estimates higher or lower; (2) proportion of HSV-2 virus among MSM in China mainland were reported; (3) HSV-2 diagnosis was conducted in a laboratory based on a strict type-specific glycoprotein-G based assays diagnostic method or PCR method; and (4) had a sample size over 20. Exclusion criteria included: (1) not being an original manuscript, such as a review article; (2) being a guideline, correspondence, and/or conference abstract; (3) the publication population did not reside in China mainland when the study was carried out; and (4) if the same epidemiological data were printed in both English and Chinese journals, English articles were preferred. We assessed the risk of bias in each individual publication using the modified quality assessment tool for systematic reviews of observational publications (QATSO). This meta-analysis was conducted by using R software. Due to extensive heterogeneity between various publications, we employed a random effect model to calculate the composite prevalence and corresponding 95% confidence intervals. We then conducted meta-regression to investigate the potential causes of observed heterogeneity. Lastly, we employed subgroup analysis based on characteristics of studies to compare the prevalence estimates across the groups. Publication bias was evaluated by funnel plot, Begg’s test and Egger’s test. Sensitivity analysis was also performed by removing each single study separately.

**Results:**

This study included 31 articles (9 published in English and 22 in Chinese) in our meta-synthesis. The pooled prevalence of HSV-2 among MSM in China mainland was 0.094 (95%CI:0.074 to 0.116). Prevalence of HSV-2 among MSM in Southwest China was higher than other regions, prevalence of HSV-2 among MSM that recruited from VCT (Voluntary Counseling and Testing) was lower than other ways, respectively. Compared to 2000–2010, the prevalence of HSV-2 among MSM in mainland China showed a downward trend during 2011–2020, however, the difference was not statistically significant .

**Conclusion:**

Prevalence of HSV-2 among MSM in China mainland is high, around 0.094. It indicated HSV-2 needed to be screening for MSM population among China mainland and proper actions should be taken to curve the trend of HSV-2 among MSM in China.

*Trial registration* CRD42020180361.

## Introduction

Herpes Simplex Virus Type 2 (HSV-2), the one of the most common causes of genital ulcers, is a sexually transmitted infection of global concern [1, 2]. It estimated that the virus has infected more than 491.5 million people aged 15–49 worldwide, accounted for 13.2% in the global population of 3735.6 million people 15–49 years of age in 2016. [3] The infection is lifelong and usually asymptomatic, with persistent reactivation and subclinical shedding, that increase its transmission potential, resulting in higher prevalence than other STIs in both the general and higher-risk population. [4–7] Prevalence of HSV-2 among men who have sex with men (MSM) in China is very high. In this population, the prevalence was 7.8% in Jiangsu Province, 14% in Shenzhen ,24.7% in Chengdu city and up to 48.6% in HIV-positive MSM population. [8–11].

Evidence suggested that HSV-2 increases the risk of HIV acquisition and transmission, [12, 13]and may have contribute to driving larger HIV epidemics [14]. HIV-HSV-2 co-infection increased transmissibility of HIV-1 and progression to AIDS [15]. Specifically, it has increased plasma HIV viral load [16–19] to a clinically significant level of 0.5 log10 copies/ml [20, 21]. It has been associated with reduced HIV-specific CD8 + T cell responses and systemic immune activation [15]. Severity of symptomatic HSV-2 has shown a correlation with low CD4 counts [22]. Several clinical trials investigated efficacy of using HSV-2 suppression as a strategy to prevent HIV transmission and to slow down HIV disease progression [20].

In May 2016, the World Health Assembly (WHA) adopted a global health sector strategy on sexually transmitted infections (STIs) for 2016–2021, which adopted alongside linked global health sector strategies on HIV and viral hepatitis, to eliminate STIs as a main public health concern by 2030 through integration of preventive and control measures. Considering that controlling HSV can also have a beneficial effect on the transmission of HIV at the same time, [23] the detection and intervention in HSV-2 infection in MSM is urgent, not only to detect HSV infection early and then take effective treatment, but also to reduce the ability of HIV transmission and help control the HIV epidemic in this population. Understanding the prevalence of HSV-2 infection in the population, especially among MSM population, is the first step in developing intervention strategies. Unfortunately, there is a paucity of data on the national magnitude of HSV-2 among MSM in China mainland. Against this background, we intend to conduct a systematic review and meta-synthesis to determine how prevalent HSV-2 infection is among MSM in mainland China. This may be the first systematic review regarding this topic.

## Method

### Review protocol and registration

This systematic review followed the recommendations of the PRISMA statement where relevant. The protocol was filed with the International Prospective Register of Systematic Reviews (PROSPERO reference CRD42020180361).

### Data sources and search strategy

Our team searched for all studies reporting the prevalence of HSV-2 among MSM population in Mainland China by utilizing PubMed, Embase, Chinese National Knowledge Infrastructure, WanFang Database for Chinese Periodicals and VIP Database for Chinese Technical Periodicals from their inception up to 28 April 2022. The search strategy is presented in Appendix 1. We did not restrict the search by language.

### Inclusion and exclusion criteria

We considered publications to be eligible for inclusion if they satisfied these conditions: (1) publication participants were MSM in China mainland. Studies were excluded if participants were exclusively all HIV-positive MSM, all HIV-negative MSM, injection-drug users, or MSM sex workers. These studies would have introduced selection bias and skewed pooled prevalence estimates higher or lower; (2) proportion of HSV-2 virus among MSM in China mainland were reported; (3) HSV-2 diagnosis was conducted in a laboratory based on a strict type-specific glycoprotein-G based assays diagnostic or PCR method; and (4) had a sample size over 20. Exclusion criteria included: (1) not being an original manuscript, such as a review article; (2) being a guideline, correspondence, and/or conference abstract; (3) the publication population did not reside in China mainland when the study was carried out; and (4) if the same epidemiological data were printed in both English and Chinese journals, English articles were preferred.

### Selection of articles and data extraction

We imported all search results using Endnote X9, identified duplicates, and excluded them. We then filtered the titles and abstracts of remaining records for relevance by two independent authors (SHI and CHEN). The full texts of potentially relevant records were then assessed for eligibility. We based our judgement to include publications on previously defined inclusion and exclusion criteria. If the two reviewers disagreed on whether or not to include a particular publication, its fate was decided by a third party (ZHANG). The two reviewers (SHI and CHEN) extracted data from relevant publications. The following was obtained from each paper: first author, publication year, study period, region, area, age, sample size, positive number, HSV-2 detecting method, study design, sampling procedure. Again, disagreements were settled by investigator three (ZHANG). We entered this information into an Excel spreadsheet.

### Quality assessment

We conducted a thorough quality-related assessment of the relevant papers incorporated by the meta-analyses utilizing the modified quality assessment tool for systematic reviews of observational publications (QATSO). The original QATSO tool comprises five quality-related sections: external validity (sampling strategy used), reporting (response rate and objectivity of measurement), confounding factors, bias (privacy), and a final, cumulative score based on the aforementioned parameters. The primary health outcome of focus in this meta-synthesis was prevalence of HSV-2. We did not assess confounding in this analysis, as the publications did not offer adaptable information on the risks for HSV-2 prevalence. Two reviewers (SHI and CHEN) conducted quality assessment independently. If the two independent reviewers disagreed on an individual publication, a third reviewer (ZHANG) made the decision.

### Statistical analysis

#### Summary findings for prevalence of HSV-2 among MSM in mainland China

We carried out the meta-synthesis of proportions by using R 4.2.0 statistical software. Heterogeneity across the studies was assessed using Cochran’s Q test, and heterogeneity was considered to be present when p < 0.05. The degree of heterogeneity was assessed using the I^2^ statistic. The I^2^ values of 25%, 50%, and 75% were considered as low, moderate, and high degrees of heterogeneity, respectively. Pooled prevalence and 95% CI of HSV-2 among the MSM population in China mainland was calculated using a random-effects model if heterogeneity was present and a fixed-effects model if heterogeneity was absent. Single raw prevalence was transformed via the Freeman-Tukey Double arcsine method to stabilize variances, all estimates were presented after back transformation.

#### Subgroup meta-analysis and meta regression for potential factors of heterogeneity

We conducted subgroup analysis and meta-regression to explore potential sources of heterogeneity by area (Central China, Eastern China, Southern China, Northern China, Southeast China, Southwest China, Northeast China, Northwest China and Multi-region), sampling procedure (Snowball, Venue-based, Respondent-driven, VCT, Time-locating, STD clinic, multiple), study design (Cross-sectional, Cohort), study period (2000–2010, 2011–2020) and QATSO (Satisfactory, Good).

#### Publication bias and sensitivity analysis

The publication bias was assessed using Begg’s test and Egger’s test and visually inspecting the funnel plot. To examine whether single study had a disproportionally excessive influence, sensitivity analysis was also conducted, in which 1 study at a time was removed and the others analyzed to estimate whether the result could have been affected markedly by a single study. P < 0.05 was considered statistically significant.

## Results

### Search and selection of studies

**Fig. 1:**
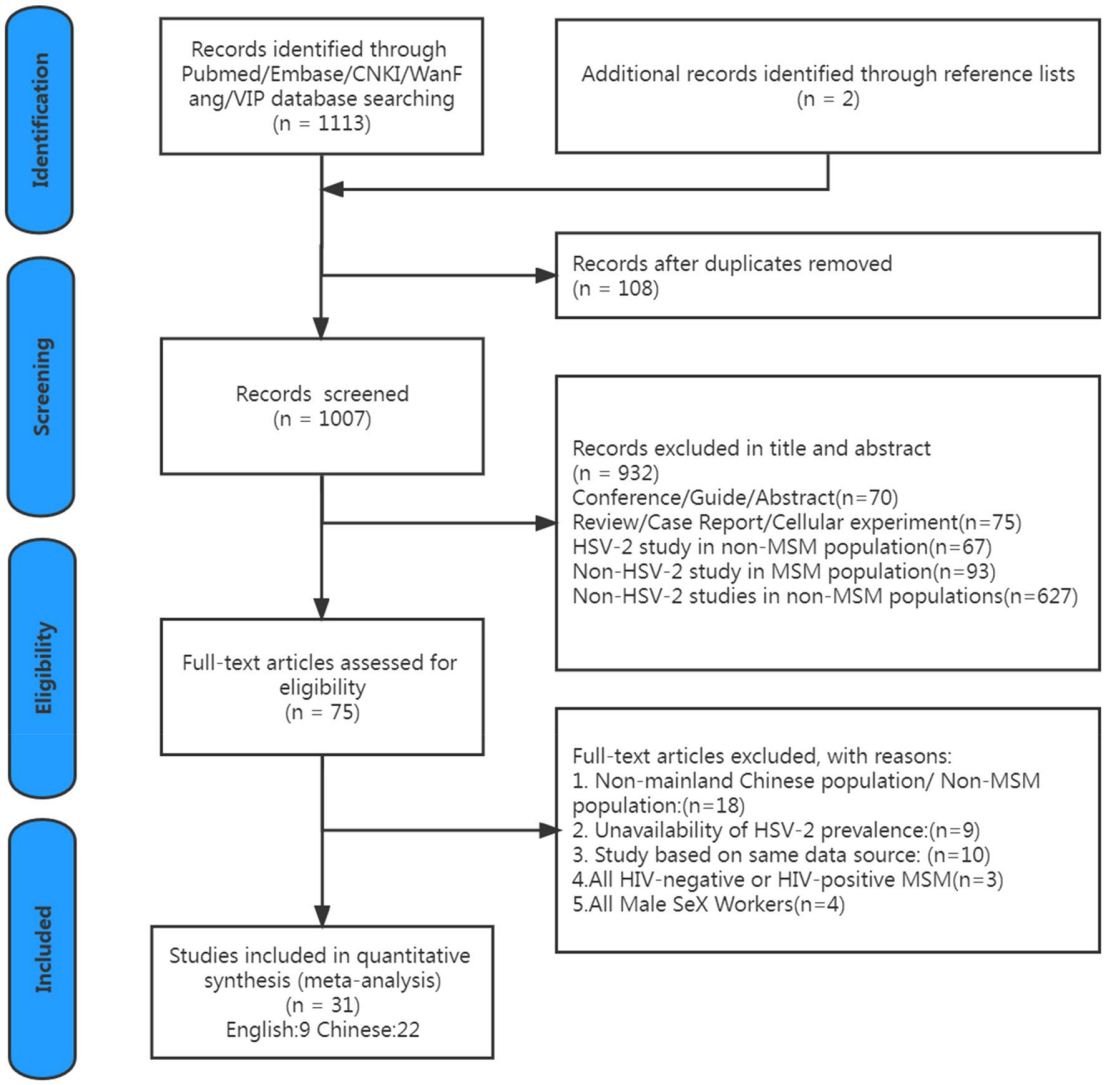
Flow chart of articles selection for systematic review

The comprehensive search for published epidemiological researches into HSV-2 among MSM conducted on the China mainland yielded 1115 hits, of which 108 were duplicates. Thus, 1007 publications were screened for titles and abstracts. These articles went through two stages of screening. Firstly, we precluded the articles failed to meet the inclusion criteria by reading the titles and abstracts. After conducting titles and abstracts screening, we identified 75 full-text articles for detailed review. When papers were excluded, it was primarily because the participants of study were not relevant population, or that it is unavailable to separate the prevalence rate of HSV-2 or studies based on same data source. Finally, we included 31 articles (9 published in English and 22 in Chinese) in our meta-synthesis. A flowchart of the selected publications is presented in Fig. 1.

### Characteristics of the publications

Table 1 shows the characteristics of included studies in this review. 31 articles published from 2006 to 2021, 9 published in English and 22 published in Chinese, including 24,858 MSM, with the largest sample size of 4415 and the smallest one of 73. The studies covered 14 provinces or municipalities, in fact, nearly 40% of the studies were conducted in the prosperous eastern China regions such as Shanghai, Shandong, Jiangsu, Anhui and Zhejiang respectively and 16% of studies were conducted in Southern China. The majority (n = 28) of studies were cross-sectional study. The majority of participants in the included studies were predominantly under 35 years. In terms of HSV-2 detection methods, only one study applied molecular method to detect HSV-2 DNA, the remaining studies used antibody detection. The studies involved a variety of recruitment methods, such as MSM venues, non-governmental organizations (NGOs) and voluntary counseling and testing (VCT). Non-probability sampling methods were employed in these studies, such as snowball sampling and respondent-driven. In the quality assessment, 10 of the included studies were considered “good” quality (values between 67 and 100%), 21 were considered “satisfactory” (values between 33 and 66%), and none were considered “poor” (values between 0 and 33%).

**Table 1:** The descriptive characteristics of included studies

First Author	Published Year	Study Period	Study location	Area	Age	Sample Size	Positive	HSV-2 Testing	Study Design	Sampling Procedure	QATSO
Xi Chen [24]	2015	2009.08-2009.11	Changsha	Central China	≥ 16, 83% under 35 years	826	113	Antibody	Cross-sectional	Venue-based, Snowball, internet	Satisfactory
Yingying Ding [25]	2017	2014.05-2014.12	Shanghai	Eastern China	18–45 87.33% under 35 years	243	23	Antibody	Cross-sectional	NGO^a^, VCT^b^	Good
Yuji Feng [10]	2010	2007.03-2007.07	Chengdu	Southwest China	16–45 Median age:24 57.5% under 25 years	538	133	Antibody	Cross-sectional	Snowball	Good
Zhenqiu Liu [26]	2017	2013.12-2014.12	Shanghai	Eastern China	Mean age: 34.09(SD = 9.85) 51.6% under 30 years	333	67	Antibody	Cross-sectional	Venue-based	Satisfactory
Junjie Xu [27]	2016	2012.06-2013.06	Shanghai, Nanjing, Changsha, Zhengzhou, Ji’nan, Shenyang, Kunming	Multi-region	≥ 18, 77.5% under 35 years	4415	552	Antibody	Cross-sectional	Internet, Venue-based, Peer referrals	Good
Hongjing Yan [28]	2016	2008.05-2008.08, 2012.09-2012.12	Nanjing	Eastern China	≥ 18, 66.3%(2008),58.7%(2012) under 30 years	1019	137	Antibody	Cross-sectional	Respondent-driven	Good
Yueping Yin [29]	2012	2009.07-2010.05	Shenzhen, Guangzhou, Changzhou	Southern China	Mean age:30.14 Median age:29 Age range: 18–66 59.6% Under 35 years	1462	234	Antibody	Cross-sectional	STD^c^ clinic, Health center, Venus-based	Satisfactory
Ning Zhao [30]	2019	2018.03-2018.10	Shenyang	Northeast China	≥ 18, 77% Over 25 years	183	2	PCR	Cross-sectional	VCT	Satisfactory
ShaSha Mao [9]	2021	2012,2014, 2016,2018	Shenzhen	Southern China	Mean age:31.5(SD = 8.38) 70.2% Under 35 years	1695	268	Antibody	Cross-sectional	Time-location sampling	Good
Ningxiao Cao [31]	2006	2003.03-2003.07	Jiangsu (No city specified)	Eastern China	Mean age:32.68(SD = 10.6) Age range:19–77 66.66% Under 35 years	90	7	Antibody	Cross-sectional	Venue-based	Satisfactory
Xianbin Ding [32]	2010	2008.02-2008.06	Chongqing	Southwest China	Mean age:26.3(SD = 7.1) Age range:18–67 64.7% Under 30 years	743	25	Antibody	Cross-sectional	Snowball	Satisfactory
Aiping Fan [33]	2017	2015.05-2016.04	Taian	Eastern China	Mean age:21.1(SD = 1.8) Age range:17–27	127	4	Antibody	Cross-sectional	VCT	Satisfactory
Jie Gao [34]	2013	2010.10-2010.12	Dehong	Southern China	Not available	88	3	Antibody	Cross-sectional	Internet, Venue-based	Satisfactory
Yanjie Gao [35]	2012	2009.08-2012.12	Beijing	Northern China	Median age:27 Age range:18–71	962	51	Antibody	Cross-sectional	Internet, Peer referrals	Satisfactory
Xiuyun Han [36]	2015	2013.04-2013.06	Ji’nan	Eastern China	Age range:17–59 85% Under 40 years	400	37	Antibody	Cross-sectional	Internet, Peer referrals	Satisfactory
Yin Han [37]	2020	2015,2016, 2017	Ji’nan	Eastern China	≥ 16, Mean age:30.21(SD = 9.38) Age range:16–73 84.1% Under 40 years	1300	73	Antibody	Cross-sectional	Snowball	Satisfactory
Guanghua Lan [38]	2013	2009–2010	Nanning	Southern China	≥ 18, Mean age:28.1	291	35	Antibody	Cohort	Snowball	Good
Pai Liu [39]	2013	2008.09-2009.02	Nanjing, Yangzhou, Wuxi, Changzhou, Suzhou	Eastern China	Mean age:30.48 Age range:18–67	388	38	Antibody	Cross-sectional	Venue-based	Satisfactory
Ying Liu [40]	2017	2015.03-2015.08	Shanghai	Eastern China	≥ 18, Mean age:29.4 83.9% Under 35 years	732	41	Antibody	Cross-sectional	VCT	Good
Chunru Lu [41]	2019	2015.06-2018.06	Shenzhen	Southern China	≥ 18, 71.19% Under 30 years	1604	34	Antibody	Cross-sectional	STD clinic	Satisfactory
Yanmin Ma [42]	2016	2014.12-2015.01	Zhengzhou	Central China	Mean age:31.6(SD = 9.4) Age range:17–76 82.87% Under 40 years	467	53	Antibody	Cross-sectional	Snowball	Good
Xiangdong Min [43]	2013	2012.07-2012.12	Kunming	Southwest China	≥ 16, Mean age:29 Age range:19–38	458	71	Antibody	Cross-sectional	Internet, Peer referrals	Satisfactory
Ji Peng [44]	2020	2019.02-2019.09	Changsha	Central China	≥ 18, 95.67% Under 40 years	462	14	Antibody	Cross-sectional	Respondent-driven	Satisfactory
Ou Qin [45]	2013	2009.07-2009.09	Guiyang	Southwest China	≥ 18, Mean age:25.8(SD = 6.5) 58.06% Under 25 years	341	22	Antibody	Cross-sectional	Snowball	Satisfactory
Huiqin Ren [46]	2012	NR	Urumqi	Northwest China	≥ 18	300	14	Antibody	Cohort	Snowball	Good
Jue Wang [47]	2012	2009.05-2009.07	Beijing, Shanghai, Kunming, Guiyang. Chongqing, Chengdu, Urumqi, Nanning	Multi-region	≥ 18 Age range:18–69	3227	344	Antibody	Cross-sectional	Snowball	Satisfactory
Hongyi Wei [48]	2014	2009.05-2012.05	Shenyang	Northeast China	Age range:17–75 59% Under 30 years	307	54	Antibody	Cross-sectional	STD clinic	Satisfactory
Zongze Xie [49]	2021	2019.01-2019.12	Taizhou	Eastern China	≥ 18, 67.14% Under 30 years	837	45	Antibody	Cross-sectional	Internet, Venue-based	Satisfactory
Yu Zhang [50]	2021	2018.10-2019.08	Guiyang	Southwest China	Age range:15–66 84.9% Under 30 years	577	74	Antibody	Cross-sectional	Respondent-driven	Satisfactory
Liangjia Zhou [51]	2016	2013.04-2013.08	Nanjing	Eastern China	Not available	370	62	Antibody	Cohort	Snowball, Peer referrals	Satisfactory
Weiming Zhu [52]	2008	2007.11-2007.12	Taizhou	Eastern China	Mean age:27.9(SD = 6) Age range:18–48 67.9% Under 30 years	73	11	Antibody	Cross-sectional	Convenient, Venue-based	Good

^a^Non-Governmental Organizations

^b^Voluntary Counseling and Testing

^c^Sexually Transmitted Disease

### The pooled prevalence of HSV-2 among MSM in Mainland China

**Fig. 2:**
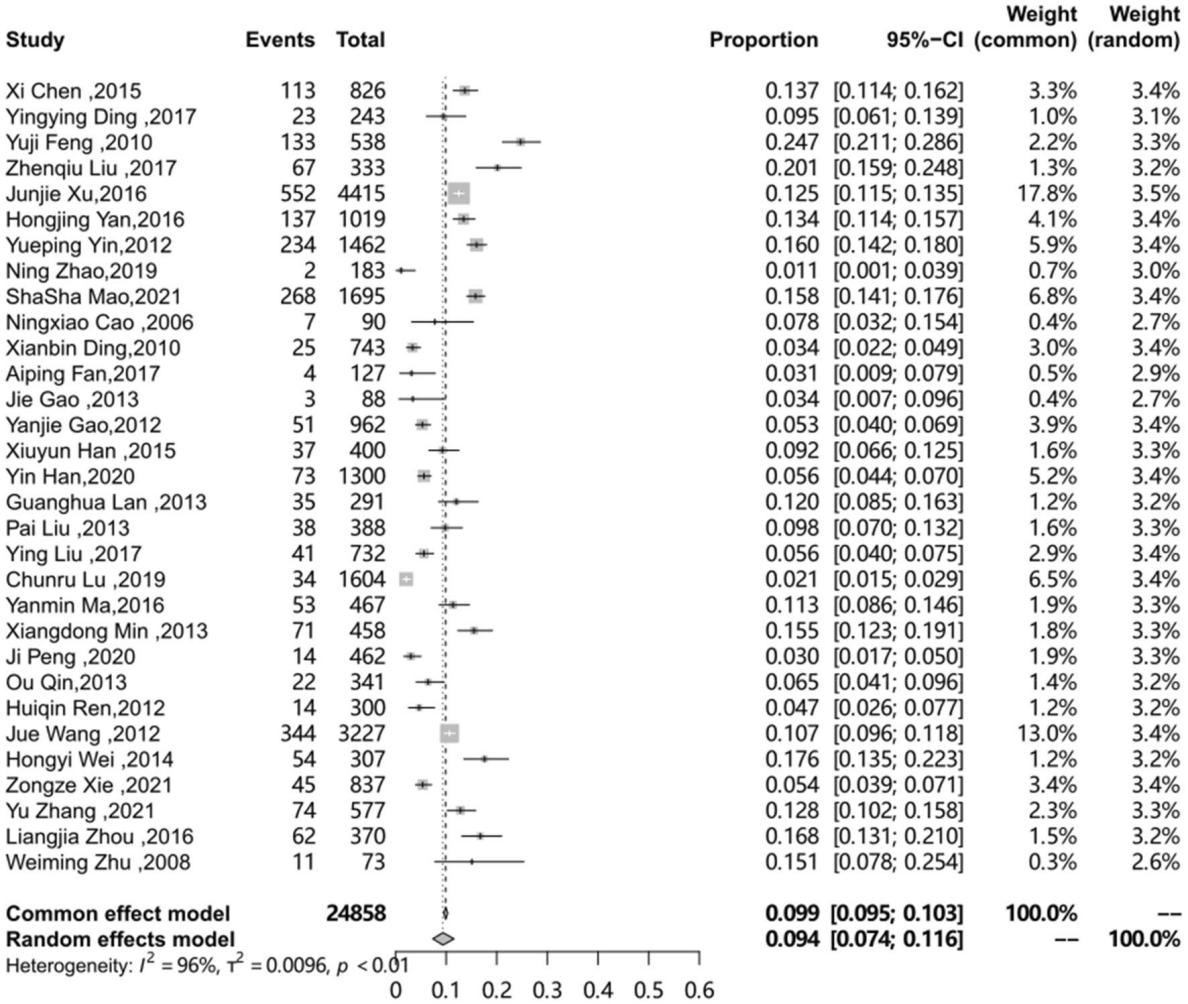
Pooled prevalence of HSV-2 among MSM in mainland China

Prevalence estimates of HSV-2 among MSM in mainland China ranged from 0.011 to 0.247, with the most estimates between 0.05 and 0.15. The random-effects pooled prevalence was 0.094 (95% confidence intervals: 0.074–0.116) with high heterogeneity (I^2^ = 96.2%, Q = 779.56, P < 0.01) among the 31 studies. The forest chart is illustrated in Fig. 2.

### Subgroup analysis

**Fig. 3:**
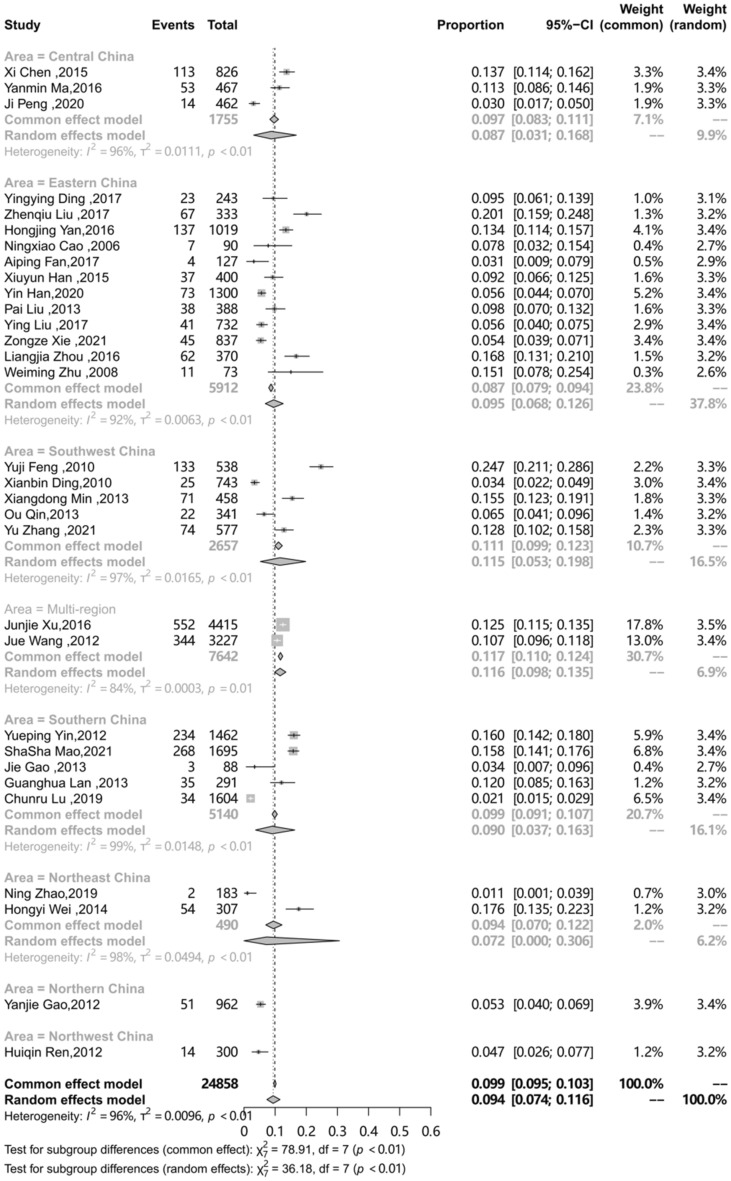
Pooled prevalence of HSV-2 among MSM in mainland China according to Area

**Fig. 4:**
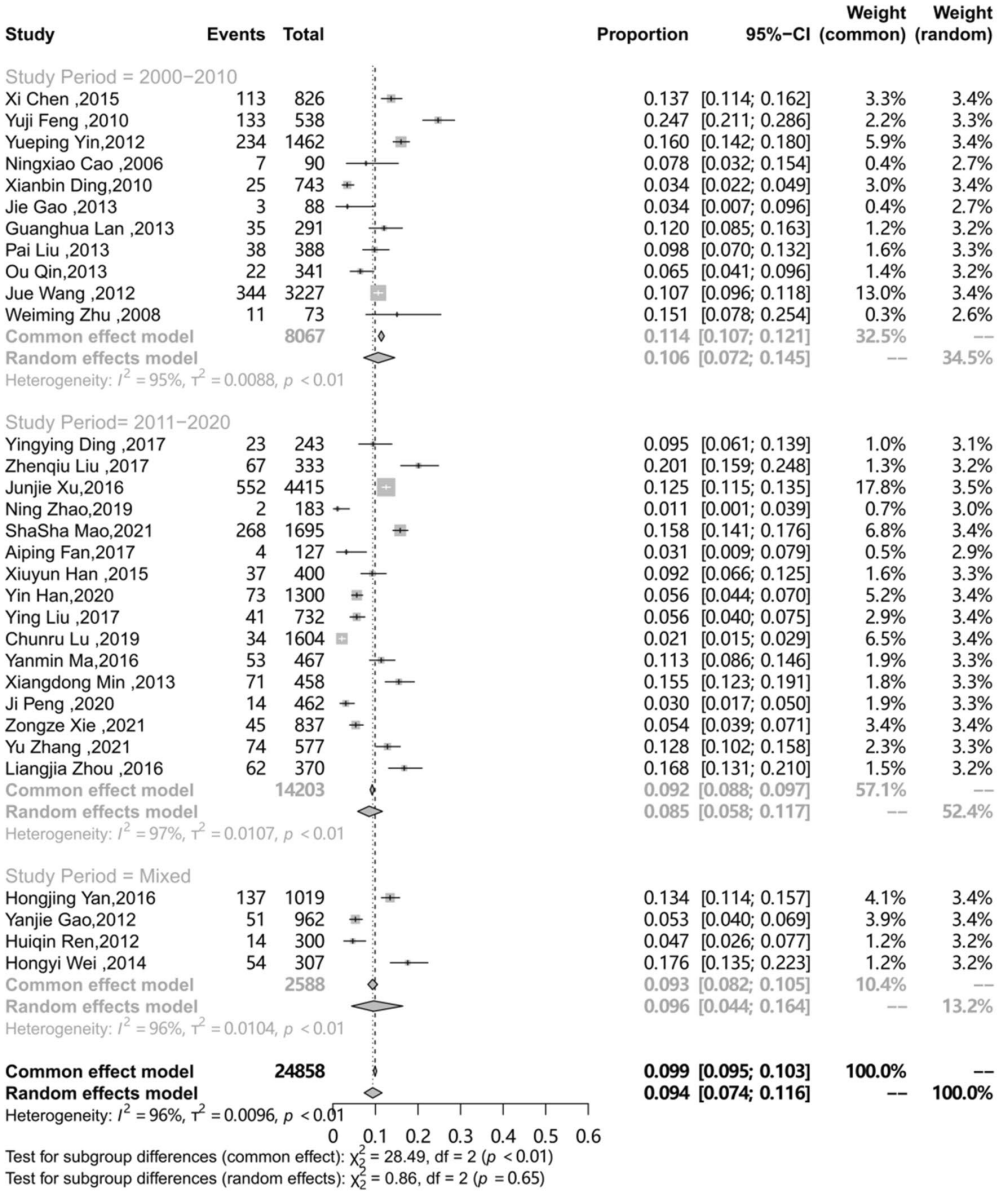
Pooled prevalence of HSV-2 among MSM in mainland China according to Study period

**Fig. 5:**
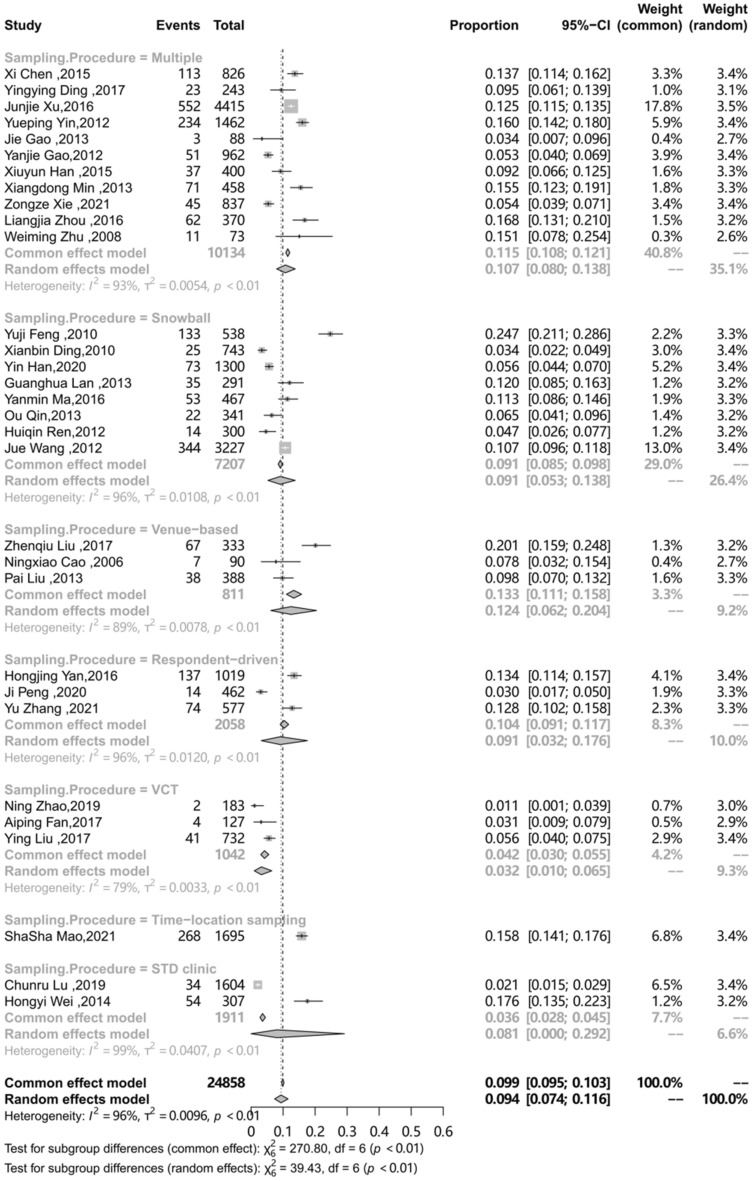
Pooled prevalence of HSV-2 among MSM in mainland China according to Sampling Procedure

**Fig. 6:**
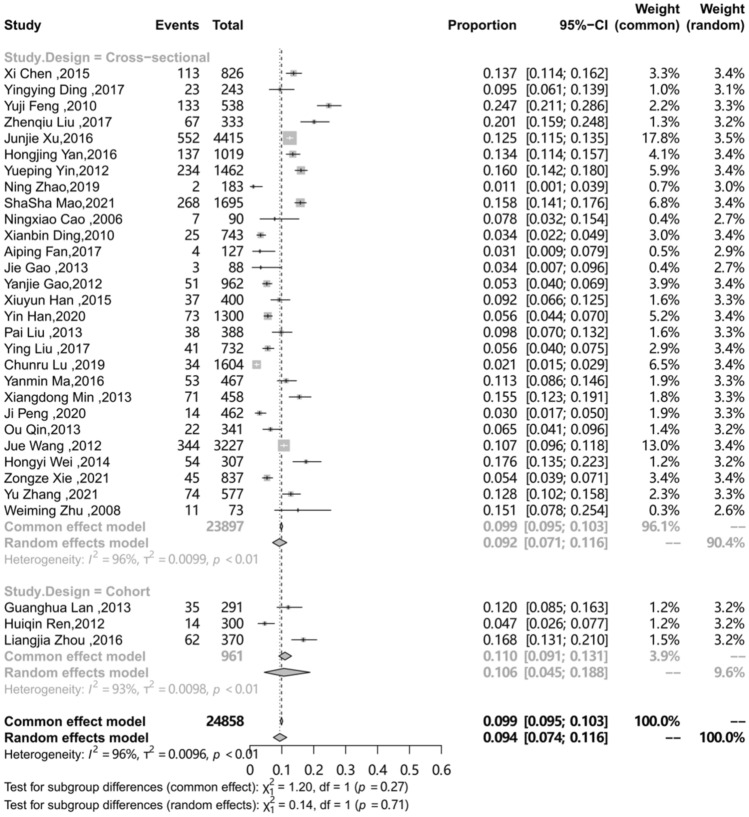
Pooled prevalence of HSV-2 among MSM in mainland China according to Study design

**Fig. 7:**
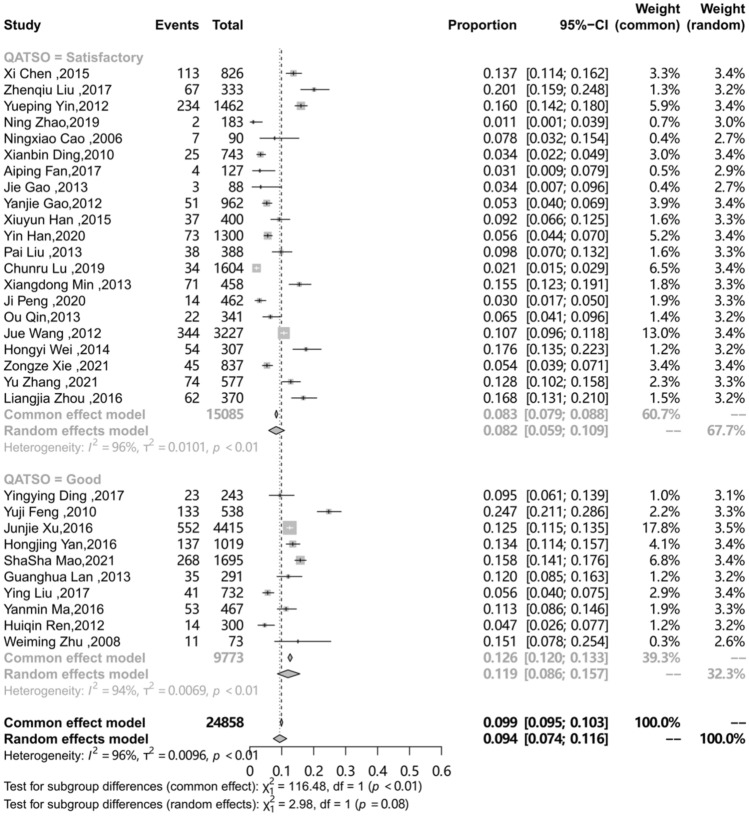
Pooled prevalence of HSV-2 among MSM in mainland China according to QATSO

The differences in prevalence reported in these studies may be due to differences in characteristics of the target population and the methodology. To explore the sources of heterogeneity, an analysis on a subgroup of area, sampling procedure, study design, study period and QATSO was performed. (Table 2) The prevalence of HSV-2 among MSM in mainland China in different regions was different (P < 0.01), with the highest in Southwest China (0.115) followed by Eastern China (0.095), Southern China (0.09), Central China (0.087), Northeast China (0.072), Northern China (0.053) and Northwest China (0.047).The subgroup analysis on the sampling procedure showed the prevalence of HSV-2 among MSM in mainland China recruited by Time-location sampling (0.158) was the highest, followed by Venue-based (0.124), multiple method (0.107), Respondent-driven sampling (RDS) (0.091) and Snowball (0.091), STD clinic (0.081), and VCT showed the lowest prevalence (0.032), respectively (p < 0.01). Compared to cohort studies, the pooled prevalence of HSV-2 among MSM was showed lower of cross-sectional studies (0.106 vs. 0.092, p = 0.71 ). The different quality of study showed different prevalence of HSV-2 among MSM in mainland China (0.082 vs. 0.119), however, the result showed no statistically significant (P = 0.08). Compared with 2000–2010, the prevalence of HSV-2 among MSM in mainland China during 2011–2020 decreased (from 0.106 to 0.085), but showed no statistically significant (p = 0.65). The subgroup analysis results presented in Table 2, forest plots for subgroup analysis are shown in Figs. 3, 4, 5, 6 and 7.

**Table 2:** Subgroup analysis of prevalence of HSV-2 among MSM in mainland China

Category	Subgroup	NO. of Studies	Prevalence (95%CI)	N	I^2^(%)	P-value for between groups
Area	Central China [24, 42, 44]	3	0.087(0.031–0.168)	1755	96	< 0.01
	Eastern China[25, 26,28,31,33,36, 37,39 ,40,49,51,52]	12	0.095(0.068–0.126)	5912	92	
	Southwest China [10, 32, 43, 45, 50]	5	0.115(0.053–0.198)	2657	97.5	
	Southern China [9, 29, 34, 38, 41]	5	0.09(0.037–0.163)	5140	98.7	
	Northeast China [30, 48]	2	0.072(0–0.306)	490	97.8	
	Northern China [35]	1	0.053(0.04–0.069)	962	-	
	Northwest China [46]	1	0.047(0.026–0.077)	300	-	
	Multi-region [27, 47]	2	0.116(0.098–0.135)	7642	83.8	
Study period	2000–2010[10, 24, 29, 31, 32, 34, 38, 39, 45, 47, 52]	11	0.106(0.072–0.145)	8067	94.9	0.65
	2011–2020 [9,25–27,30,33,36,37,40−44,49−51]	16	0.085(0.058–0.117)	14,203	96.9	
	Mixed [28, 35, 46, 48]	4	0.096(0.044–0.164)	2588	95.6	
Sampling procedure	Multiple [24, 25,27,29,34–36,43,49,51,52]	11	0.107(0.08–0.138)	10,134	93.3	< 0.01
	Snowball[10, 32, 37, 38, 42, 45–47]	8	0.091(0.053–0.138)	7207	96.3	
	Venue-based [26, 31, 39]	3	0.124(0.062–0.204)	811	89.1	
	Respondent-driven [28, 44, 50]	3	0.091(0.032–0.176)	2058	96.3	
	VCT [30, 33, 40]	3	0.032(0.01–0.065)	1042	78.6	
	Time-location sampling [9]	1	0.158(0.141–0.176)	1695	-	
	STD clinic [41, 48]	2	0.081(0–0.292)	1911	98.8	
Study Design	Cohort [38, 46, 51]	3	0.106(0.045–0.188)	961	92.7	0.71
	Cross-sectional [9,10,24−37,39–45,47−50,52]	28	0.092(0.071–0.116)	23,897	96.4	
QATSO	Good [9, 10, 25, 27, 28, 38, 40, 42, 46, 52]	10	0.119(0.086–0.157)	9773	93.6	0.08
	Satisfactory [24,26,29–37,39,41,43–45,47 –51]	21	0.082(0.059–0.109)	15,085	96.2	

## Meta-regression test, publication Bias, and sensitivity analysis

**Fig. 8:**
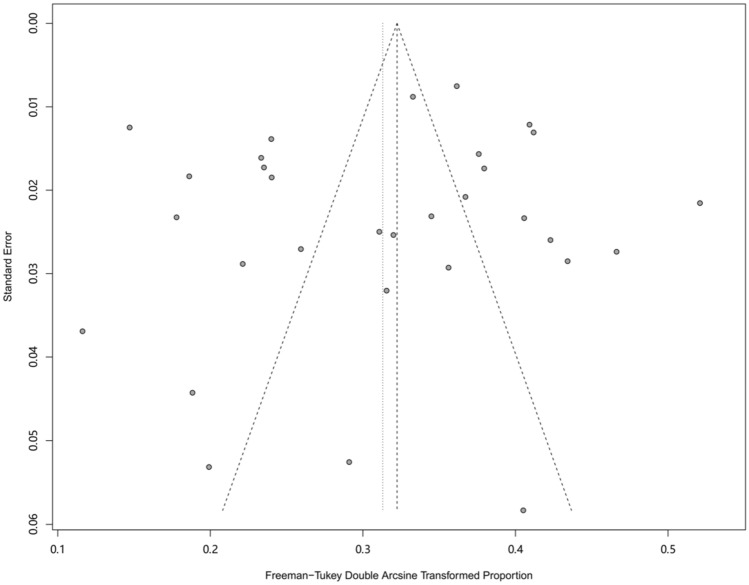
Funnel plot for publication bias of the prevalence of HSV-2 among MSM in mainland China

**Fig. 9:**
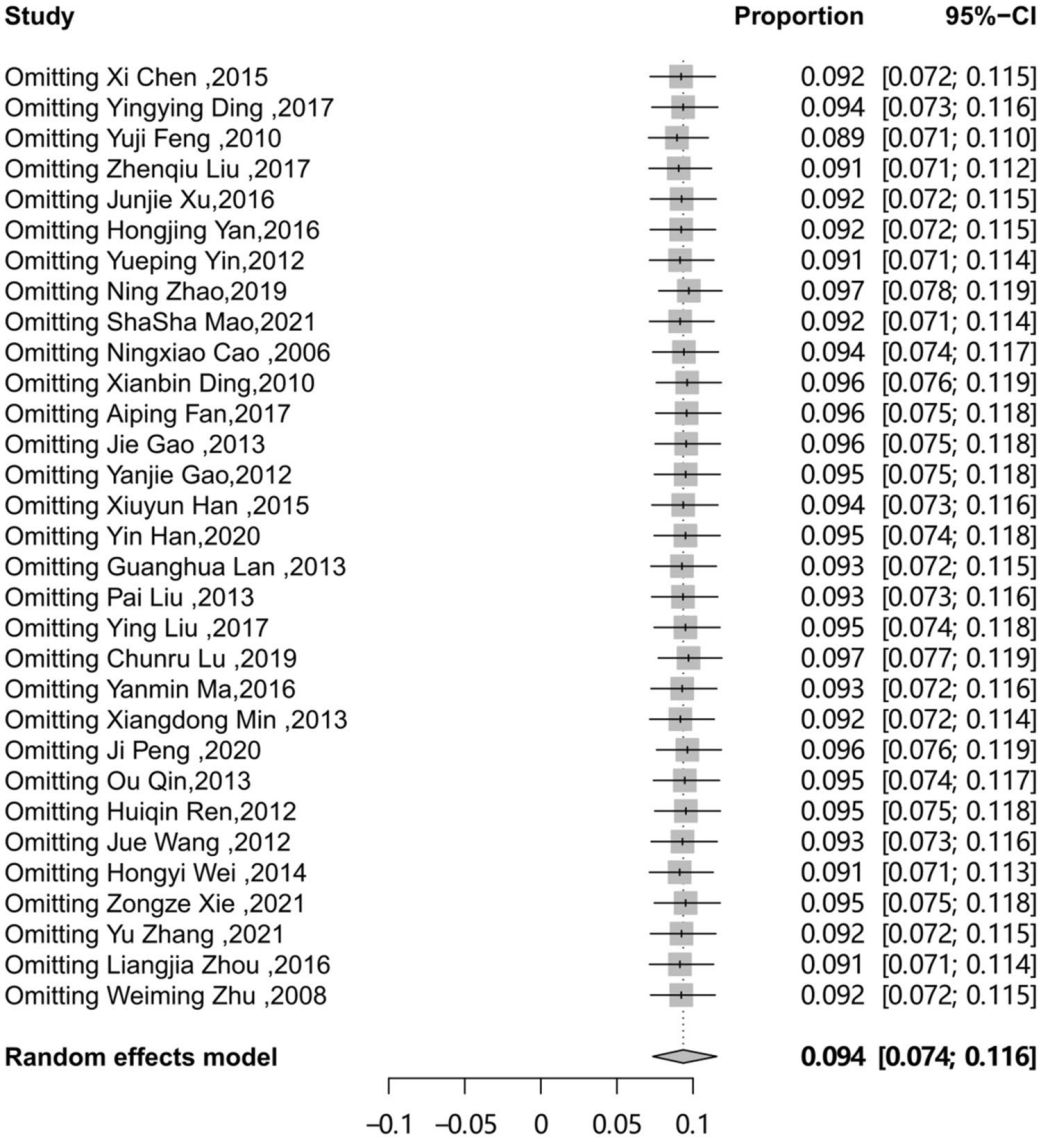
Sensitivity analysis diagram of the prevalence of HSV-2 among MSM in mainland China

We conducted a meta-regression analysis to explore the potential heterogeneity among studies. We included the following factors into the meta-regression model: area, sampling procedure, study design, study period and QATSO. The result indicated that sampling procedure impacted the estimation of point prevalence (p < 0.05). However, the result didn’t fully clarify the high level of heterogeneity. (Table 3). We found no significant publication bias in the 31 studies through the funnel plot (Fig. 8), the Egger’s test and Begg’s test showed same conclusion (Egger: t = − 0.62 P = 0.537, Begg: z = − 0.42 P = 0.671). Finally, we investigated the influence of a single study on the overall prevalence of HSV-2 by excluding one study at a time. The pooled prevalence of HSV-2 among MSM were consistent and without apparent fluctuation, with a range from 0.089 (95% CI: 0.071 to 0.110) to 0.097 (95% CI: 0.077 to 0.119) (Fig. 9). This analysis confirms the stability of our result.

**Table 3:** Results of Meta-regression analysis for prevalence of HSV-2 among MSM in mainland China

Covariate	Meta-regression coefficient	95%CI	P value
Area			
Central China [24, 42, 44] (Ref.^a^)			
Eastern China [25,26,28,31,33,36,37,39,40,49,51,52]	0.016	− 0.124 to 0.155	0.825
Southwest China [10, 32, 43, 45, 50]	0.047	− 0.111 to 0.204	0.562
Southern China [9, 29, 34, 38, 41]	0.007	− 0.151 to 0.165	0.932
Northeast China [30, 48]	− 0.023	− 0.222 to 0.177	0.824
Northern China [35]	− 0.068	− 0.315 to 0.179	0.590
Northwest China [46]	− 0.08	− 0.332 to 0.172	0.533
Multi-region [27, 47]	0.046	− 0.149 to 0.241	0.644
Study period			
2000–2010 [10, 24, 29, 31, 32, 34, 38, 39, 45, 47, 52](Ref.)			
2011–2020 [9,25–27,30,33,36,37,40−44,49−51]	− 0.037	− 0.117 to 0.043	0.367
Mixed [28, 35, 46, 48]	0.019	− 0.137 to 0.099	0.755
Sampling procedure			
Multiple [24,25,27,29,34–36,43,49,51 ,52](Ref.)			
Snowball [10, 32, 37, 38, 42, 45–47]	− 0.028	− 0.118 to 0.063	0.546
Venue-based [26, 31, 39]	0.028	− 0.103 to 0.158	0.679
Respondent-driven [28, 44, 50]	− 0.028	− 0.153 to 0.098	0.667
VCT [30, 33, 40]	− 0.152	− 0.282 to − 0.022	0.022
Time-location sampling [9]	0.074	− 0.125 to 0.272	0.467
STD clinic [41, 48]	− 0.049	− 0.198 to 0.099	0.512
Study design			
Cohort [38, 46, 51](Ref.)			
Cross-sectional [9,10,24−37,39–45,47−50,52]	− 0.023	− 0.146 to 0.099	0.713
QATSO			
Good [9, 10, 25, 27, 28, 38, 40, 42, 46, 52](Ref.)			
Satisfactory [24,26,29–37,39,41,43–45,47−51]	− 0.062	− 0.136 to 0.013	0.106

*a: Reference group*

## Discussion

Thus far, there is a lack of pooled estimation regarding the prevalence of HSV-2 among MSM population in mainland China and even the results of different studies vary greatly. This study therefore aimed to determine, through a systematic review and meta-analysis, the prevalence of HSV-2 among the MSM population in mainland China. Our systematic review of the observational study included 31 studies that involved a total of 24,858 participants in mainland China and covered 14 provinces or municipalities of the country. The aggregate prevalence of HSV-2 among MSM in mainland China was 0.094, suggested that nearly one of ten MSM have infected HSV-2 in mainland China. This result was higher than the study reported in general populations in mainland China [53]. When compared with the prevalence in other countries, our estimate prevalence was moderate, lower than Tanzania (22.7%) [54], Peru (46.3%) [55] and America (26.1%) [56]. The prevalence of HSV-2 among MSM was found to be low in this study compared to other countries or regions, which may be related to the economy, culture, etc. However, the absolute number of MSM with HSV-2 infection in China’s booming population was still large. Action is still needed to prevent and control the spread of HSV-2 in MSM population.

In view of the extreme heterogeneity observed, subgroup analysis of the prevalence of HSV-2 among MSM was conducted on different characteristics and obtain some information from analysis. The prevalence of HSV-2 among MSM varied from region to region. Specially, the prevalence of HSV-2 among MSM in Southwest China was higher than the other regions. The Open-Door Policy in 1979 not only brings globalization to the China economy, but also has an impact on the sexuality of the Chinese population. Homosexuality that was once obscured by the contemporary Chinese society also emerged to become a legitimate lifestyle choice [57]. Southwest China includes several areas with a high HIV prevalence, such as Chongqing, which is a city that is accepting towards homosexuality and has very open attitudes about sex [58]. However, MSM used condoms on a regular basis was founded lower than the national average level in this region, which may lead increasing the likelihood of disease transmission. [59]Considering the differences in HSV-2 infection rates within different regions, each region needs to conduct an in-depth analysis for its own region to find individualized causes and take targeted measures. Our study illustrated that, the prevalence of HSV-2 among MSM in mainland China obtained by VCT was lower than other groups, which was because that only those with definite awareness and attention to STDs would come for the voluntary counseling and testing, therefore, this segment of the population is also more likely to do a better job of self-protection and self-health monitoring. Finally, we have also studied the trend of HSV-2 prevalence among MSM populations, dividing the studies into two groups based on study period, 2000–2010 and 2011–2020. Compared with 2000–2010, it showed a slight decrease in HSV-2 infection in the MSM population between 2011 and 2020, however, the difference was not statistically significant. It can be assumed that HSV-2 infection in the MSM population has remained relatively stable over the two decades. This suggests that HSV-2 infection in the MSM population is not receiving enough attention. Whether from the perspective of preventing HSV-2 infection or enhancing HIV prevention through HSV-2 prevention, policy makers need to pay adequate attention to HSV-2 infectious and develop appropriate policies to reduce its epidemic in the MSM population.

In our estimation, this is the first meta-synthesis regarding the prevalence of HSV-2 among the MSM population in mainland China. This publication was, however, vulnerable to several shortcomings. First, both subgroup and meta-regression analyses provided limited explanation of heterogeneity. This suggests that there may be other factors that could explain the differences between studies. This may also be related to the specificity of the MSM population, which is still not widely accepted in China, although its acceptance has increased, making it a relatively hidden population and therefore difficult to obtain a sample through probability sampling, as well as a complex group where multiple factors such as age, income and marital status. All these details are not available through existing studies, which may lead us to conclude that there is no source of heterogeneity in the results based on the available material. This also poses a challenge for our future research efforts; it is expected to make use of probability sampling or conduct further accurate research on MSM population with a certain demographic characteristic. Second, there are more cross-sectional and fewer longitudinal publications of HSV-2 infection among MSM, which cannot describe incidence of HSV-2 among MSM. Also, as the publications included in this meta-synthesis only covered 14 provinces and municipalities in mainland China, there was a distinct lack of data from other provinces and municipalities. Therefore, the results we ascertained may not be applicable to all geographic areas of China. However, this publication covered most parts of the county, so the results are still reliable. Furthermore, we could not discern the survey population’s age, which rendered additional analysis of the age-based connections impossible.

## Conclusion

This meta-analysis provides a comprehensive synthesis of HSV-2 prevalence in literature targeting Chinese MSM population. The study concluded that MSM are at particularly high risk of contracting HSV-2 infection in China’s mainland. Sustainable, holistic, and efficacious prevention efforts must be implemented for assisting this vulnerable population. Additional, comprehensive epidemiological examinations should be conducted to acquire a more exact estimate of the status of HSV-2 infection epidemics among the MSM population in China.

## Data Availability

All data generated or analyzed during this study are included in this published. article.

## Appendix 1. Search strategies for traditional database

We conducted a comprehensive search in database of PubMed, Embase, Chinese National Knowledge Infrastructure (CNKI), WanFang Database for Chinese Periodicals, and the VIP Database for Chinese Technical Periodicals. using a combination of Medical Subject Headings and free text including terms related to HSV-2, MSM, prevalence and China mainland. All related published papers from database’s inception to April 28,2022 were identified and subsequently stored using EndNote X9.

**Table:**

No.	Query	Results
PubMed		
#1	‘Herpesvirus 2, Human’[MeSH Terms] OR ‘Herpes Simplex Virus Type 2’ OR ‘Human Herpesvirus 2’ OR ‘HHV-2’ OR ‘HSV-2’ OR ‘Herpes Simplex Virus 2’	9121
#2	‘Homosexuality, Male’[MeSH] OR ‘men who have sex with men’ OR ‘MSM’ OR ‘homosexual men’	29,391
#3	‘China’ [MESH] OR ‘China’ OR ‘Chinese’ OR ‘mainland’	2,537,478
#4	‘Prevalence’ [MeSH Terms] OR ‘epidemiology’ [MeSH Terms] OR ‘prevalence’ OR ‘epidemiology’ OR ‘incidence’	3,860,450
#5	#1 and #2 and #3 and #4	25

**Table:**

No.	Query	Results
EMBASE		
#1	‘herpes simplex virus 2’/exp OR ‘herpes simplex virus 2’ OR ‘herpes simplex virus type 2’ OR ‘human herpesvirus 2’ OR ‘hhv-2’ OR ‘hsv-2’ OR ‘herpes simplex virus 2 antibody’	14,748
#2	‘homosexuality’/exp OR ‘homosexuality’ OR ‘men who have sex with men’/exp OR ‘men who have sex with men’ OR ‘msm’ OR ‘homosexual men’/exp OR ‘homosexual men’	64,323
#3	‘China’/exp OR ‘china’ OR ‘chinese’ OR ‘mainland’	3,026,167
#4	‘prevalence’/exp OR ‘prevalence’ OR ‘epidemiology’/exp OR ‘epidemiology’ OR ‘incidence’	5,841,916
#5	#1 and #2 and #3 and #4	37

**Table:**

No.	Query	Results
CNKI		
#1	SU%=(‘单纯疱疹病毒’ + ‘单纯疱疹’ + ‘HSV’ + ‘HSV-2’ ) OR FT=(‘单纯疱疹病毒’ + ‘单纯疱疹’ + ‘HSV’ + ‘HSV-2’ )	71,705
#2	SU%=(‘男同’ +’男同性恋’ + ‘男男性行为’ + ‘同性恋’ + ‘男男同性恋’ ) OR FT=(‘男同’ +’男同性恋’ + ‘男男性行为’ + ‘同性恋’ + ‘男男同性恋’ )	70,755
#3	SU%=(‘流行’ + ‘患病率’+ ‘趋势’ + ‘现状’ + ‘疾病流行’ + ‘疾病负担’ + ‘感染’) OR FT=(‘流行’ + ‘患病率’+ ‘趋势’ + ‘现状’ + ‘疾病流行’ + ‘疾病负担’ + ‘感染’)	22,796,140
#4	#1 and #2 and #3	877

**Table:**

No.	Query	Results
WanFang		
#1	主题:(“单纯疱疹病毒” or “单纯疱疹” or “HSV”or “HSV-2” ) or 题名或关键词:(“单纯疱疹病毒” or “单纯疱疹” or “HSV”or “HSV-2”)	28,578
#2	主题:(“男同” or“男同性恋” or “男男性行为” or “同性恋” or “男男同性恋” ) or 题名或关键词:(“男同” or“男同性恋” or “男男性行为” or “同性恋” or “男男同性恋” )	12,022
#3	主题:(“流行” or “患病率”or “趋势” or “现状” or “疾病流行” or “疾病负担” or “感染” ) or 题名或关键词:(“流行” or “患病率”or “趋势” or “现状” or “疾病流行” or “疾病负担” or “感染”)	6,497,557
#4	#1 and #2 and #3	35

**Table:**

No.	Query	Results
Chongqing VIP		
#1	M=单纯疱疹病毒 OR U=单纯疱疹病毒 OR M=单纯疱疹 OR U=单纯疱疹 OR M = HSV OR U = HSV OR M = HSV-2 OR U = HSV-2	12,890
#2	M=(男同 OR 男同性恋 OR 男男性行为 OR 同性恋 OR 男男同性恋 ) OR U=(男同 OR 男同性恋 OR 男男性行为 OR 同性恋 OR 男男同性恋)	445,581
#3	M=(流行 OR 患病率 OR 趋势 OR 现状 OR 疾病流行 OR 疾病负担 OR 感染) OR U=(流行 OR 患病率 OR 趋势 OR 现状 OR 疾病流行 OR 疾病负担 OR 感染)	71,652,709
#4	#1 and #2 and #3	139
